# Effect of User Charges on Secondary Level Surgical Care Utilization and Out-of-Pocket Expenditures in Haryana State, India

**DOI:** 10.1371/journal.pone.0125202

**Published:** 2015-05-04

**Authors:** Deepak Balasubramanian, Shankar Prinja, Arun Kumar Aggarwal

**Affiliations:** School of Public Health, Post Graduate Institute of Medical Education and Research, Sector 12, Chandigarh, 160012, India

## Abstract

**Background:**

Generation of resources for providing health care services is an important issue in developing countries. User charges in the form of Surgical Package Program (SPP) were introduced in all district hospitals of Haryana to address this problem. We evaluate the effect of this SPP program on surgical care utilization and out-of-pocket (OOP) expenditures.

**Methods:**

Data on 25437 surgeries, from July 2006 to June 2013 in 3 districts of Haryana state, was analyzed using interrupted time series analysis to assess the impact of SPP on utilization of services. Adjustment was made for presence of any autocorrelation and seasonality effects. A cross sectional survey was undertaken among 180 patients in District hospital, Panchkula during June 2013 to assess the extent of out of pocket (OOP) expenditure incurred, financial risk protection and methods to cope with OOP expenditure. Catastrophic health expenditure, estimated as any expenditure in excess of 10% of the household consumption expenditure, was used to assess the extent of financial risk protection.

**Results:**

User charges had a negative effect on the number of surgeries in public sector district hospitals in all the 3 districts. The mean out-of-pocket expenditure incurred by the patients was Rs.4564 (USD 74.6). The prevalence of catastrophic expenditure was 5.6%. A higher proportion among the poorest 20% population coped through borrowing money (47.2%), while majority (86.1%) of those belonging to richest quintile paid from their monthly income or savings, or had insurance.

**Conclusion:**

There is a need to increase the public financing for curative services and it should be based on the needs of population. Any form of user charge in public sector hospitals should be removed.

## Introduction

India has a surgical rate of 369 per 100,000 population and total number of surgeries in the range of 37,04,446–44,38,792. Thus, it has a large share of the burden of surgical diseases [1]. Moreover, the share of healthcare and medicine expenses is 5.5% of the total household expenditure [2]. So the high disease burden translates to soaring financial costs. This combination leads to worsening of existing poverty [3]. The problem is compounded by the lack of protective mechanisms [4,5]. There are a number of calls to universalizing health care in India (HLEG, 12^th^ Five Year Plan) [6], however resource generation remains a major issue [7].

In resource constrained low-income countries, introduction of user charges is justified based on their potential to generate resources for public sector [8]. The Surgical Package Program (SPP) was also one such program to levy user charges for surgical care services in public sector hospitals in the entire state of Haryana. These user charges were levied at utilization of surgical services at district hospitals. Below poverty line (BPL) population and those living in urban slum were exempted from any user charge. Certain surgeries such as caesarean sections; eye surgeries for adults and children under general anesthesia; and cleft lip surgeries were also exempted from SPP charges. The user charge covered pre-surgical medicine, diagnostics, and cost of surgery and post-surgical medicines upto 14 days following discharge.

Out-of-pocket payments are defined in the World Health Report 2010 as “charges or fees levied for consultations with health professionals, medical or investigative procedures, medicines and other supplies, and for laboratory tests levied by government, nongovernmental organizations, faith-based and private health facilities. They are sometimes officially sanctioned charges and sometimes unofficial or so-called ‘under-the-table’ payments” [9]. Coming under its purview are coinsurance, co-payment and deductibles paid by the insured [10]. User fees refer to the official fees collected by public health facilities [11]. Thus, they are a subset of out-of-pocket expenditure.

Global experience suggests that user charges result in decrease in utilization of services [8]. However, much of the work on this subject is drawn from the developed country setting, where near-universal coverage of health care services has been achieved [12–14]. In most of these settings, the charges were levied in the form of mix of some co-payment or coinsurance or deductible. The major purpose reported for this form of demand-side cost sharing is to discourage frivolous use of health care services and with overall aim of reducing health care costs.

Evidence does exist from the developing countries as well [11,15,16]. However there are 2 main deficiencies in the existing evidence. Firstly, majority of the existing studies do not have control areas to assess impact. Secondly, most of these studies had only a pre and post design with single overall observation for both periods. Finally, most of the evidence from developing countries is to assess impact of user charges on primary care services, and none for surgical services in particular. Moreover, price elasticity for utilization of care is different for primary and secondary level services. In India, previous attempts at introduction of user charges in Haryana resulted in a decline in inpatient service utilization [17]. There has been no assessment of the Haryana Government’s introduction of user charges for surgical care at hospitals providing secondary care. We aim to bridge this gap in evidence by assessing the impact of user charges program (SPP) on utilization of secondary level surgical services and out-of-pocket expenditures in Haryana state of India. We use robust time series design, and incorporating 3 different districts which were at different levels of utilization for surgical services.

## Methods

### Study Design and District Selection

Haryana state, which has a population of 25,351,462, comprises of 21 districts. Three districts in Haryana state of north India were chosen using purposive sampling. We attempted to include diverse type of districts for assessing the impact, varying from one with highest to lowest baseline levels of utilization. Our study was based at secondary hospitals as the new form of user charges under the Surgical Package Program were levied at this level. Two high performing districts (Panchkula and Rohtak) and one lowest performing district (Gurgaon) were selected (Table 1). Districts Panchkula, Rohtak and Gurgaon are henceforth referred to as District 1, 2 and 3 respectively. For the category with highest levels of utilization of surgical services, we included 2 districts—one with high and low levels of rural population. This was considered important, as there are wide geographic variations in utilization of health care services. All district hospitals are located in urban areas, which could systematically bias the utilization upwards in districts with a higher proportionate urban population. While district 1 district has 44.2% rural population, nearly 58% of district 2 population belonged to rural area. Regional stratification was particularly chosen within the high performing districts in view of this differential access to urban-based district hospitals which has been reported to be influenced by the area of residence of patient [18].

**Table 1:** Profile of Study Districts Based on Socio-economic and Demographic indicators.

S.No	Indicator	Panchkula	Rohtak	Gurgaon
1	Population (number)	558890	1058683	151085
2	Literacy rate (%)	83.4	80.4	84.4
3	Number of Community Health centres	2	7	2
4	Number of Primary health centre	10	23	13
5	Number of sub-centre	51	114	76
6	Health personal in the district hospital (number)	179	118	145
7	Population below age 6 (%)	13.8	14.3	19.9
8	Under-Five mortality (%)	97.9	96.3	115.6
9	Female Literacy (%)	69	63.2	48.3
10	Households using Safe drinking water (%)	90.4	66.8	80.7
11	Households having toilet facility (%)	56.4	45.9	42.1
12	Composite Development Index (number)	0.74723	0.68889	0.48243
13	Development District Rank in Haryana (number)	2	5	19
14	Development District Rank in India (number)	190	221	543

District hospitals in India are the hub of provision of secondary care services in India, with a hospital present in each district. A total of 24049 Primary health centres and 148366 sub-centres provide primary health care services in India [19]. Cases requiring specialist care are referred to a Community Health Centre (CHC) or a district hospital. Since there is acute shortage of specialist doctors at CHCs—only a quarter of these CHCs have a gynecologist; district hospitals provide the bulk of secondary health care services [19]. The 722 district hospitals in India have a bed strength varying from 75 to 500 beds depending on the population, size and terrain of the district [19]. Besides offering basic specialty services (outpatient care, inpatient care and emergency services), newborn care, psychiatric services, physical medicine and rehabilitation services, accident and trauma services, and anti-retroviral therapy are provided. The manpower varies from 117 medical, paramedical and administrative personal in 100 bedded hospital to 422 in a 500 bedded hospital [20].

### Intervention: Surgical Package Program

The Surgical Package Program was introduced in July 2009 can be seen as a measure to introduce co-payment to increase revenue of district hospitals. Patients have to pay a one-time fee at the time of admission for all the services. The cost of each surgery was fixed and uniform in each district. In this arrangement, hospital purchases and provides all medicines, consumables, diagnostics etc. and patient is not supposed to incur any further OOP expenditure beyond the fixed co-payment. The user charges for surgery in these public sector hospitals were almost one-third to half of the charges levied in private sector for similar surgeries. The user charge covered pre-surgical medicine, diagnostics, cost of surgery and post-surgical medicines upto 14 days following discharge. Certain surgeries such as Caesarean sections, eye surgeries for adults, and children under General anaesthesia; and cleft lip surgeries at all public hospitals were provided free of charge. Below poverty line (BPL) population and those living in urban slum were exempted from user charge. Since the public hospital would become monopsonistic purchaser, it was foreseen that the total cost of surgery would be reduced.

### Study Design and Data Collection

Month-wise data on the number of surgeries performed at district hospitals was collected from the districts for the period from July 2006 to June 2013. Data for district 3 was collected from January 2007 to June 2013 in view of non-availability of data on surgeries during initial six months of pre-intervention period. The date of initiation of SPP program in Haryana state was July 2009.

A cross-sectional survey was conducted during the month of June 2013 in the district hospital of district 1. A sample size of 155 was considered appropriate, assuming a mean out-of-pocket expenditure incurred by the households during hospitalization to be INR 5000 (standard deviation of INR 5000, lowest mean of INR 4000), power of 80% and 95% confidence interval [21]. Considering a failure to response rate of 10%, the final sample size was calculated to be 170 patients. These patients were selected by sequential sampling. All patients who were registered in the SPP during the study period, present in the hospital and who consented were enrolled. Patients who were registered in the SPP, but failed to avail the services; those undergoing cataract surgery or cesarean section (as they are free of cost under national health programs); and those who were operated in emergency during night and did not stay in the hospital till the morning or died were excluded. A total of 31 (17%) patients were excluded on these grounds. Overall, 180 patients were enrolled for the study.

To assess the out-of-pocket (OOP) expenditure incurred by the patient, a structured questionnaire developed by the National Sample Survey Organization to collect data on OOP expenditure was adapted for use in present study [21]. The patient was interviewed on the day of admission and followed up daily to collect data on OOP expenditure during the last 24 hours in the hospital. Hence, we do not expect any recall bias. Monthly household expenditure was estimated as the sum of the expenditure on food, clothing, shelter, education, medical expenses, personal effects, fuel and miscellaneous items during the last month prior to hospitalization. The direct OOP expenditure was assessed by collecting data on on the OOP for surgery package, medicines (apart from the surgery package charges), diagnostics (apart from the surgery package charges), money spent on travel and food. The indirect OOP expenditure was calculated from the loss of income due to days of hospitalization. The patients had to recall their expenses for the past month.

### Data Analysis

An interrupted time series (ITS) analysis was done using month-wise data on number of surgeries. Cataract surgeries which were already free such as those under the National Blindness Control Programme [22] were excluded from the number of surgeries. Segmented linear regression, with 1^st^ July 2009 as the intervention period, was used. Durbin-Watson test was used to check for autocorrelation and the models were adjusted for seasonality by differencing method. If the Durbin-Watson test showed significant positive autocorrelation (Durbin-Watson statistic substantially <2) at a particular lag, differencing was done at that particular lag. The Beta coefficients of the trend before & after intervention and change in level were noted along with their direction (positive or negative). The β_*0*_ estimates the number of surgeries per month at time zero, β_*1*_ estimates the change in the number of surgeries per month that occurred with each month before the intervention, β_*2*_ estimates the level change in the number of surgeries per month immediately following the intervention and β_*3*_ estimates the change in the number of surgeries per month that occurred each month after the intervention [23]. All analysis was done using SPSS v21.0 and Minitab 17 statistical software.

Data on OOP expenditure from the cross-sectional survey of 180 patients was analyzed to present mean levels of expenditure. OOP expenditure was stratified in terms of medicines, diagnostics etc was analyzed to assess the components of the expenditure. Expenditure incurred for different type of surgeries was analyzed. Catastrophic health expenditure was defined as households spending more than 10% of the total household expenditure as OOP on healthcare [24]. This definition was followed, as our data did not permit differentiating between food and non-food consumption expenditure. This definition is also endorsed for application in Indian context by the draft National Health Policy 2015 of India.

### Ethical Issues

The study was approved by the Institute Ethics Committee of the Post Graduate Institute of Medical Education and Research, Chandigarh. Administrative approval was sought from the State Health Department of Haryana, and medical officer incharge of each district hospital which was enrolled in the study for collecting data. Written informed consent was obtained from all the patients enrolled in the study.

## Results

### Impact of surgical package program on utilization of services

**Fig 1:**
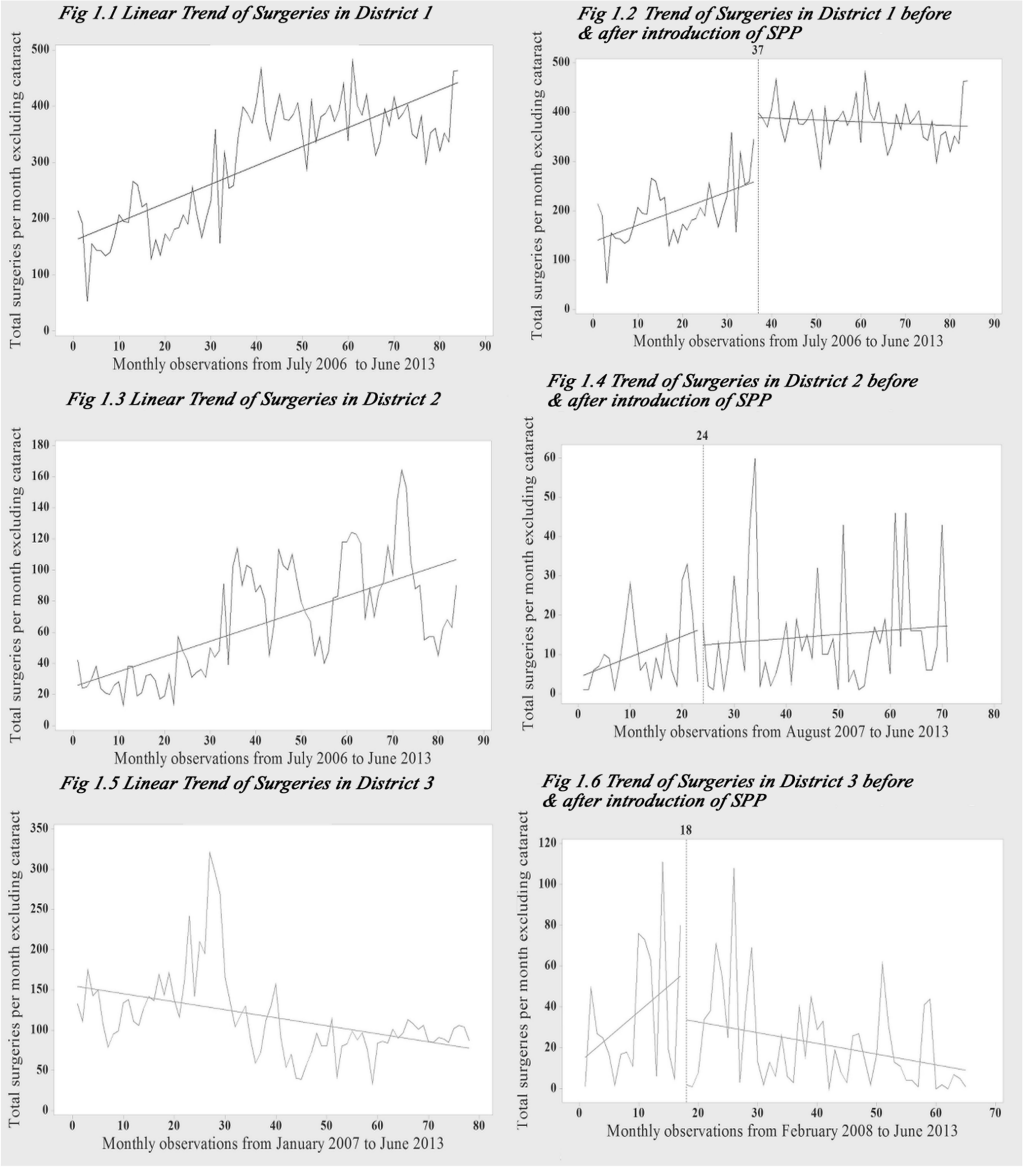
Trend of surgical care utilization, before and after the introduction of user charges in districts Panchkula, Rohtak and Gurgaon. Month-wise data on the number of surgeries performed at district hospitals was collected from the districts for the period from July 2006 to June 2013 in Panchkula, Rohtak, and from January 2007 to June 2013 for Gurgaon. Fig 1.2: Unadjusted model was chosen for Panchkula, as it showed no positive autocorrelation or seasonality. Fig 1.4: The model for Rohtak was adjusted for both, first-order correlation and seasonality. Fig 1.6: The model for Gurgaon was adjusted for both, first-order correlation and seasonality.

The number of surgeries taking place per month during July 2006 to June 2013 increased from 214 to 464 in district 1, from 42 to 90 in district 2 respectively, while it decreased from 133 to 87 during January 2007 to June 2013 in district 3 (Fig 1). In district 1, SPP had a significant immediate increase in the number of surgeries. The monthly increase pre-package was significant, while after the introduction of SPP there was an insignificant decrease (Fig 1 & Table 2). There was no autocorrelation and test for seasonality was also negative.

**Table 2:** Impact of SPP on service utilization at district hospitals in Haryana.

	Parameter	Panchkula	Rohtak	Gurgaon
**Model 1**	**SLM**			
	*R* ^*2*^	0.805	0.537	0.604
	*Durbin-Watson test*	1.76	0.740 ##	1.262 ##
	*Constant (*β*0)*	137.61 (15.84)**	13.88 (8.53)	92.75 (12.15)**
	*Trend before intervention (*β*1)*	3.35 (0.75)**	1.28 (0.40)**	4.47 (0.72)**
	*Change in level (*β*2)*	131.12 (20.42)**	28.50 (11.0)**	-131.69 (15.68)**
	*Trend after intervention (*β*3)*	-0.37 (0.49)	-0.03 (0.26)	-0.04 (0.35)
**Model 2**	**SLM 1st differencing**			
	*R* ^*2*^		0.091	0.296
	*Constant (*β*0)*		2.14 (4.80)	10.99 (8.09)
	*Trend before intervention (*β*1)*		0.61 (0.23)**	1.7 (0.47)**
	*Change in level (*β*2)*		-8.12 (6.14)	-28.29 (9.89)**
	*Trend after intervention (*β*3)*		0.03 (0.15)	-0.52 (0.22)**
**Model 3**	**SLM 1st, 12th differencing**			
	*R* ^*2*^		0.096	0.243
	*Constant (*β*0)*		6.04 (6.41)	27.36 (13.67)**
	*Trend before intervention (*β*1)*		-3.52 (7.42)	-8.14 (14.80)
	*Change in level (*β*2)*		0.66 (0.47)	2.39 (1.33)
	*Trend after intervention (*β*3)*		0.16 (0.16)	-1.01 (0.28)**

**—P<0.05;

##—Durbin Watson test showing significant positive autocorrelation.

In district 2, the unadjusted model showed positive autocorrelation. After adjusting for first-order autocorrelation, followed by correction for seasonality, the SPP had a non-significant immediate decrease in the number of surgeries. The monthly change pre-package showed an insignificant decrease, while after the introduction of SPP, there was a small insignificant increase (Fig 1 & Table 2).

In district 3, the unadjusted model showed positive autocorrelation. After adjusting for first-order autocorrelation, followed by correction for seasonality, the SPP had an insignificant immediate decrease in the number of surgeries. The monthly change pre-package showed an insignificant decrease, while after the introduction of SPP, there was a significant decrease (Fig 1 & Table 2).

### Extent of out of pocket expenditure

The general characteristics of the 180 patients included in the study are summarized in Table 3. The mean total OOP expenditure incurred for surgical care was USD 74.6. An average OOP of USD 41.1 was incurred directly on user charges under SPP, while USD 13.5 USD 19.9 were other direct and indirect expenditures respectively (Table 4). Majority of the other direct OOP expenditure was for the diagnostics (38.4%). The mean hospitalization expenditure incurred by the households as a proportion of the annual consumption expenditure was 4.8% (4.2–5.4%). The mean annual health expenditure incurred by the households as a proportion of the annual consumption expenditure was 11.6% (10.5–12.6%). The poorest quintiles spent a higher proportion (7.1%) of their annual consumption expenditure on hospitalization, as compared to the richest quintiles (2.9%). Similar differential was noted for overall annual health expenditure between the poorest (16.7%) and the richest (6.8%).

**Table 3:** Background characteristics of the patients for assessment of out-of-pocket expenditures.

Characteristic	Number of patients n (%)
***Sex***	
Male	83 (46.1)
Female	97 (53.9)
***Age***	
<20 years	29 (16.1)
20–29 years	43 (23.9)
30–39 years	30 (16.7)
40–49 years	44 (24.4)
50–59 years	19 (10.6)
Above 60 years	15 (8.4)
***Education of patient***	
Illiterate	45 (25.0)
Primary school	16 (8.9)
Middle school	44 (24.4)
High School	55 (30.6)
Secondary school	13 (7.2)
Graduate	7 (3.9)
***Religion***	
Hindu	151 (83.9)
Muslim	8 (4.4)
Sikh	21 (11.7)
***Total family income per month (in rupees)***	
<Rs.Rs.5000	27 (15.0)
Rs.5001-10000	102 (56.7)
Rs.10001-15000	15 (8.3)
Rs.15001-20000	10 (5.6)
Above Rs.20000	26 (14.4)
***Insurance***	
Availed insurance	24 (13.3)
Not availed insurance	156 (86.7)
***Insurance type***	
Private	3 (12.5)
Employer based	14 (58.3)
Government (ESI, RSBY)	7 (29.2)
***Type of surgery***	
Major surgery	144 (80.0)
Minor surgery	36 (20.0)
***BPL status***	
BPL	27 (15.0)
Non-BPL	153 (85.0)
***Department***	
Surgery	117 (65.0)
Orthopaedics	21 (11.7)
Gynaecology	22 (12.2)
ENT	20 (11.1)

**Table 4:** Out-of-pocket expenditure incurred by the patients enrolled under Surgical Package Program (user charge program) in district Panchkula, Haryana.

	Mean (95% CI)	Median (IQR)
	(in rupees)	(in rupees)
***Overall***		
**Total expenditure**	4564 (4083 to 5045)	4290 (1682–6400)
**Direct OOP expenditure**		
*SPP expenditure*	2516 (2242–2790)	3000 (500–4000)
*Other expenditure*	826 (617–1035)	330 (116–836)
**Indirect costs**		
*Loss of productivity*	1222 (1039–1405)	800 (475–1500)
***General Surgery***		
**Total expenditure**	4553 (4003–5103)	4745 (1310–6722)
**Direct OOP expenditure**		
*SPP expenditure*	2677 (2304–3050)	3000 (500–5000)
*Other expenditure*	646 (510–782)	325 (110–857)
**Indirect costs**		
*Loss of productivity*	1230 (992–1468)	800 (400–1500)
***Orthopaedics***		
**Total expenditure**	5774 (3290–8258)	2917 (1725–10930)
**Direct OOP expenditure**		
*SPP expenditure*	2238 (1516–2960)	1500 (500–3500)
*Other expenditure*	2141 (737–3545)	340 (140–5165)
**Indirect costs**		
*Loss of productivity*	1395 (662–2128)	667 (367–1833)
***Gynaecology***		
**Total expenditure**	5289 (4236–6342)	5040 (4376–6109)
**Direct OOP expenditure**		
*SPP expenditure*	2932 (2289–3575)	3500 (3500–3500)
*Other expenditure*	884 (154–1614)	385 (141–689)
**Indirect costs**		
*Loss of productivity*	1473 (1046–1900)	1250 (825–1900)
***ENT***		
**Total expenditure**	2558 (1985–3131)	2858 (1225–3307)
**Direct OOP expenditure**		
*SPP expenditure*	1405 (1032–1778)	2000 (500–2000)
*Other expenditure*	437 (166–708)	165 (100–456)
**Indirect costs**		
*Loss of productivity*	716 (559–873)	683 (450–975)

Almost, 59% of the patients paid for the healthcare from their monthly income or savings, 7.8% had insurance and 31.1% had to borrow money. A higher proportion among the poorest quintiles coped through borrowing money (47.2%), while majority (86.1%) of those belonging to richest quintile paid from their monthly income or savings or had insurance. The prevalence of catastrophic expenditure was 5.6%. Among the different surgical specialities, prevalence of catastrophic expenditure was proportionately higher in Orthopaedics (19.0%). While 13.9% incurred catastrophic expenditure in poorest quintile, none from the richest quintile experienced catastrophic outcomes of OOP expenditure incurred on surgical care.

## Discussion

The burden of surgically treatable conditions is increasing, as well as the cost of healthcare, especially the share that is spent out-of-pocket. User charges in the form of a Surgical Package Program (SPP) was introduced in all district hospitals of Haryana in order to ensure generate revenue for public sector, decrease cost to the patient in comparison to private facility and also use the revenue generated to improve quality of services. Ultimately the aim was to draw patients from the private facilities to public sector. Our findings suggest that the user charges (SPP) did not have a significant increase in the number of surgeries taking place each month in the three district hospitals. The mean out-of-pocket expenditure incurred by the patients was Rs.4564, which resulted in a 5.6% prevalence of catastrophic expenditures. Almost, 31% of the patients had to take loan/borrow money to pay for the hospitalization, which rose to 47% in case of poorest households. It appears that the program could not achieve its objectives of increasing the utilization of services and reducing the out-of-pocket expenditure incurred by the patient.

This is the first study from India to comprehensively evaluate the impact of introduction of user charges on hospitals providing secondary level surgical care. Another study in the past had analyzed the effect of user charges on inpatient hospitalizations [17]. However, the user charges in the past studies were quite less as compared to those for surgical services under SPP. Secondly, the nature of data which was available in the previous study prevented use of better analytical methods for arriving at more robust conclusions. Our study assessed the effect of user charges under SPP on two different aspects—utilization of services and OOP expenditures. Secondly, there were no other significant program changes for surgical services during the intervening period, which could have confounded our results. Similarly, other factors such as socio-demographic characteristics, access to transport services, general awareness for treatment seeking etc. are also unlikely to have changed dramatically to have influenced the study results. So whatever change in utilization is observed in our study is likely to be the effect of the user charges.

Similar observation of declining trends in surgical care utilization in all 3 districts, irrespective of baseline utilization and extent of urban population, strengthens the association between imposition of user charges and declining surgical care utilization. In terms of development ranking of districts within India, Panchkula, Rohtak and Gurgaon ranked 190, 221 and 543 respectively among 591 included districts, while within Haryana they ranked 2, 5 and 19 respectively among the 21 included districts (Table 1) [25]. Hence these comprise of diverse range of districts with different developmental rank, which improves the generalizability of our findings.

User charges, i.e., where consumer pays part of the total cost of service have been widely experimented by policy makers in both developed and developing countries [12]. Most Organization for Economic Cooperation and Development (OECD) countries have introduced user charges to counter the demand side moral hazard of risk pooling mechanisms and their association with rising health care expenditures [13]. On the contrary, their introduction in public sector institutions of developing countries was necessitated by a need to generate resources for providing health care services [17]. Secondly, in most developed country context, user charges are introduced in the form of a copayment, coinsurance or deductible operating in the presence of a risk-pooling arrangement which offers near-universal or a reasonably comprehensive coverage for health care services. This rise in the copayment rates affects the group with the higher healthcare expenditures negatively [26]. However, user charge for surgical services as in the present study was introduced in a pre-existing environment of high out-of-pocket expenditures with little financial risk protection against catastrophic effects of accessing health services. A recent USAID report found that hospitalizations in Haryana state led to nearly 30% households incurring catastrophic health expenditures [27].

Most of the low and middle income countries (LMICs), including India have introduced policies which aim at reducing the extent of OOP, and thus progressing towards universal health care. Broadly, two forms of routes have been taken. In the first, it is through a risk pooling mechanism where Government pays for the community-rated premium of the poor through tax-funding and the population gets coverage for a defined benefit package. Care is cashless at point of use through a network of public or private empanelled hospitals. In this, the rich may want to opt in the insurance scheme with payment of a pre-determined premium which is usually not risk rated. The *Rashtriya Swasthya Bima Yojana* (RSBY) in India is an example of this model [28]. Again, numerous other State funded health insurance schemes in India also tend to take this model [5]. Other countries like Thailand, Ghana, Rwanda and others also have similar models [29–31]. In the second model, the Government invests adequate resources though tax-money into the public sector and provides health care services. This is a model which is publicly financed and publicly provided health care services. This model is being promoted by Sri Lanka. Some Indian states, such as Haryana, are also now attempting to emulate this model [32]. While no model is perfect, overall aim of each of these strategies is to reduce the extent of OOP expenditures and improve financial risk protection.

In India, evidence from the NSSO survey shows that about 20% and 30% of urban and rural households respectively who reported unmet need for curative care, cited financial constraint and high OOP as the reason for the same [21]. Hence strategies have to be evolved to reduce this OOP, rather than introducing other forms of copayments or user charges. The XIIth Five Year Plan and the recently released draft National Health Policy endorse this viewpoint and outline strategies for reducing OOP expenditures [33,34].”

Design of health-programme evaluations has been dominated traditionally by experimental approaches used in medicine, in which specific individuals or clusters of people receive an intervention whereas others do not. Studies tend to be undertaken in controlled environments in which the influence of external factors is kept to a minimum or eliminated. Such randomized controlled trials are considered to provide the highest quality of evidence to evaluate impact of an intervention or program. However, real world health system interventions are far from controlled experiments [35]. Moreover, the policy makers are usually interested in rolling out the program in the entire geographic area, with little chance for researchers to design studies which have better power to establish cause-effect relationship [36]. The SPP program in Haryana state was also introduced in July 2009 in the entire state.

In order to assess the impact of such programs, novel designs have to be evolved. We used month-wise data on number of surgeries to apply an interrupted time series (ITS) analysis to assess the impact of user charges. ITS designs are considered as robust methods to study intervention effects in a non-randomized setting. As compared to cross-sectional design which compares outcomes between groups at a single point in time and pre-post designs which compare estimates at two time point, ITS uses multiple observations over time, both prior and subsequent to an intervention, helping to control for existing trends and study the dynamics of intervention effect improving the validity of results [23]. As illustrated in Ramsay et al (2003), ITS design allows for the statistical investigation of potential biases in the estimate of the effect of the intervention [37]. These potential biases include secular trend—the outcome may be increasing or decreasing with time; cyclical or seasonal effects—there may be cyclical patterns in the outcome that occur over time; or duration of the intervention—the intervention might have an effect for the first three months only after it was introduced and in such case yearly data would not have identified this effect; and random fluctuations—these are short fluctuations with no discernible pattern that can bias intervention effect estimates. Another major problem with the conventional ordinary least squares (OLS) regression approach is that the critical assumption of independent errors can be violated by the presence of autocorrelation. Autocorrelation means that the errors (or residuals) of the fitted model are correlated with each other at particular time lags.

In order to circumvent these biases in causal attribution of intervention effect, segmented linear regression (SLR) analysis and Auto-Regressive Integrated Moving Average (ARIMA) modelling based on the Box–Jenkins methodology using interrupted time series data have been recommended [37]. Lagarde (2012) recommends SLR over ARIMA, grading former technique better on the basis of less data requirement, and having an explanatory approach than predictive [38]. Hence we believe that our choice of statistical modelling methods is well justified. We also found evidence of auto-regression which we controlled in the model.

Finally, we attempted to include diverse type of districts for assessing the impact, varying from one with highest to lowest baseline levels of utilization. Utilization of services has been measured as the number of surgeries (major & minor). For the category with highest levels of utilization of surgical services, we included 2 districts—one with high and low levels of rural population. This was considered important, as there are wide geographic variations in utilization of health care services. All district hospitals are located in urban areas, which could systematically bias the utilization upwards in districts with a higher proportionate urban population.

We would like to note certain methodological limitations in our study. The rate of surgical care utilization could not be calculated due to lack of a well-defined base population of a district hospital. The patients in a given district hospital may not be necessarily confined to those living in the same district and could belong to areas of neighboring district as well. Secondly, all the patients for the cross-sectional component of study were drawn from 1-month duration. However, there is no evidence to suggest seasonality in surgical care. Some seasonal pattern in occurrence of births and hence utilization of obstetric care is noted [39], however, such patients were excluded from both the OOP expenditure analysis. We would like to note a limitation in our analytical approach. We acknowledge that it is best to collect detailed data on consumption expenditure which can be stratified into food and non-food expenditure. This can enable calculation of catastrophic health expenditures as the percent of those households which incur an expenditure on health care in excess of 40% of household’s non-food expenditure. However, detailed collection of food and non-food expenditure was not possible in hospital setting as patient comfort needs to be taken into account. So, we considered 10% of the total expenditure as the threshold for catastrophic expenditure [4]. This has been put forth by Pradhan [24], and is endorsed for use in Indian context by the country’s recent draft National Health Policy 2015 [34].

The result of the interrupted time series analysis shows that the post-slope in each of the three districts, Panchkula, Rohtak and Gurgaon, was less that the pre-slope. In high-performing districts, user charge stopped the pace of increase in the number of surgeries, while in low-performing district there was a declining trend. Overall, the user charge had a negative influence on the utilization of surgical care.

The OOP levels observed is our study comparable with that reported in NSSO 60^th^ round survey for hospitalization episode {Rs. 5300 (1925–16055)} [21]. It has been observed that poor people spend higher proportion of their monthly expenditure on health and hence, are vulnerable to impoverishment [40]. The prevalence of catastrophic expenditure in our study (5.6%) is lower than 13.9% and 14% reported by other studies using the NSS 2009–10 and NSS 2004–05 data [21,41].

Overall we conclude that introduction of user charges for surgical care incurs high OOP in public sector hospitals. User charges were introduced in the public sector hospital for surgical services to generate revenue, and improve quality of services. However, user charges under SPP did not have a significant increase in the number of surgeries in the three district hospitals. Rather user charges had a negative effect in all 3 districts on surgical care utilization. OOP expenditure incurred by the household remains high, which has particularly affected the lowest wealth quintile. The prevalence of catastrophic expenditure was highest in the poorest quintile. This calls for removal of any form of user charges in public sector hospitals.

There is a need to increase the public financing for curative services based on the needs of population. Financial protection mechanisms to the vulnerable population could be considered in the short run, till public financing is up scaled to the desired level. Haryana has initiated an emergency ambulance system, which has been shown to improve access to health services [42]. Moreover, it has been reported to operate efficiently [43]. We recommend that the ambulance service should also be made available free of charges to any patient who wishes to be transported to a public sector hospital. This will reduce the transportation costs and provide improved access to the public sector hospitals.

## Supporting Information

S1 Dataset (XLSX)
